# Functional evaluation of pediatric patients after discharge from the intensive care unit using the Functional Status Scale

**DOI:** 10.5935/0103-507X.20170066

**Published:** 2017

**Authors:** Gabriela Alves Pereira, Camila Wohlgemuth Schaan, Renata Salatti Ferrari

**Affiliations:** 1 Universidade Federal de Ciências da Saúde de Porto Alegre - Porto Alegre (RS), Brazil.; 2 Hospital de Clínicas de Porto Alegre, Universidade Federal do Rio Grande do Sul - Porto Alegre (RS), Brazil.; 3 Hospital Moinhos de Vento - Porto Alegre (RS), Brazil.

**Keywords:** Morbidity, Critical care, Child, Intensive care units, pediatric, Child, hospitalized, Morbidade, Cuidados intensivos, Criança, Unidades de terapia intensiva pediátrica, Criança hospitalizada

## Abstract

**Objective:**

To evaluate the functional status of pediatric patients after discharge from the pediatric intensive care unit using the Functional Status Scale and to compare the time of invasive mechanical ventilation, length of stay in the pediatric intensive care unit, and Pediatric Index of Mortality 2 results among individuals with different degrees of functional impairment.

**Methods:**

A cross-sectional study was conducted on patients who were discharged from a pediatric intensive care unit. The functional evaluation by the Functional Status Scale was performed on the first day after discharge from the unit, and the Pediatric Index of Mortality 2 was used to predict the mortality rate at the time of admission to the pediatric intensive care unit.

**Results:**

The sample consisted of 50 individuals, 60% of which were male, with a median age of 19 [6 - 61] months. The overall score of the Functional Status Scale was 11.5 [7 - 15], and the highest scores were observed in the "motor function" 3 [1 - 4] and "feeding" 4 [1 - 4] domains. Compared to patients who were not readmitted to the pediatric intensive care unit, patients who were readmitted presented a worse overall score (p = 0.01), worse scores in the "motor function" (p = 0.01), "feeding" (p = 0.02), and "respiratory" (p = 0.036) domains, and a higher mortality rate according to the Pediatric Index of Mortality 2 (p = 0.025).

**Conclusion:**

Evaluation of the functional status using the Functional Status Scale indicated moderate impairment in patients after discharge from the pediatric intensive care unit, mainly in the "motor function" and "feeding" domains; patients who were readmitted to the pediatric intensive care unit demonstrated worse overall functional, motor function, feeding and respiratory scores. Individuals with greater functional impairment had longer times of invasive mechanical ventilation and hospitalization in the pediatric intensive care unit.

## Abstract (pt)

**Objetivo:**

Avaliar a funcionalidade de pacientes pediátricos após alta da unidade de terapia intensiva pediátrica por meio da *Functional Status Scale* e comparar o tempo de ventilação mecânica invasiva, tempo de internação e o *Pediatric Index of Mortality* 2 entre os indivíduos com diferentes graus de comprometimento funcional.

**Métodos:**

Estudo transversal, realizado com pacientes egressos de uma unidade de terapia intensiva pediátrica. A avaliação funcional pela *Functional Status Scale* foi realizada no primeiro dia após a alta da unidade, tendo sido utilizado o *Pediatric Index of Mortality* 2 como índice preditivo de mortalidade do momento da admissão na unidade.

**Resultados:**

A amostra foi composta por 50 indivíduos, sendo 60% do sexo masculino, com mediana de idade de 19 meses [6 - 61]. O escore global da *Functional Status Scale* foi de 11,5 [7 - 15] e maiores escores nos domínios "função motora" 3 [1 - 4] e "alimentação" 4 [1 - 4]. Os pacientes que reinternaram na unidade de terapia intensiva pediátrica demonstraram, comparativamente aos que não reinternaram, ter pior escore global (p = 0,01), "função motora" (p = 0,01), "alimentação" (p = 0,02), "respiração" (p = 0,036) e maior índice de mortalidade pelo *Pediatric Index of Mortality* 2 (p = 0,025).

**Conclusão:**

A avaliação da *Functional Status Scale* indicou disfunção funcional moderada dos pacientes após a alta da unidade de terapia intensiva pediátrica, principalmente na função motora e alimentação; pacientes que reinternaram na unidade de terapia intensiva pediátrica demonstraram ter piores escore funcional global e função motora, alimentação e respiração. Indivíduos com maior comprometimento funcional apresentaram maior tempo de ventilação mecânica invasiva e internação na unidade de terapia intensiva pediátrica.

## INTRODUCTION

The predictive instruments of mortality for pediatric patients have been extensively studied and used in pediatric intensive care units (PICU) in different parts of the world.^(1)^ However, survival assessment is no longer the only outcome of interest because ICU mortality has declined by approximately 2% per year since 2000.^(2)^ This reduction is mainly due to technological advances and improved care, such as the implementation of multidisciplinary work and the development of routines and protocols appropriate for patient care and safety, as well as improved communication among ICU staff, patients and their families.^(2,3)^

Due to their increased survival, critical patients remain for longer periods in the hospital environment, creating a population of chronic patients after discharge from the ICU.^(3)^ A prolonged hospital stay commonly leads to reduced functional independence due to the use of neuromuscular blockers and medications such as corticosteroids and mainly because patients are subjected to prolonged invasive mechanical ventilation (IMV).^(3)^

Symptoms presented by the survivors include psychological problems (anxiety and depression), cognitive dysfunction, worsening pulmonary function and development of peripheral neuromuscular complications.^(2)^ These limitations and disabilities presented after discharge affect the overall performance and development of the child in the physical, cognitive, emotional and/or social health dimensions^(1)^ and are related to the morbidity of these patients. These outcomes are rarely reported in the literature or evaluated in a manner that is inadequate for the pediatric age group and its specificities.^(4-6)^

The Functional Status Scale (FSS) was developed based on the concepts of Daily Life Activities (DLA) and adaptive behavior.^(7)^ This scale evaluates the functional score in motor and cognitive domains specifically developed for hospitalized pediatric patients.^(7)^ It is a quantitative, fast and reliable method, independent of subjective assessments, applicable to a wide age range, from term newborns to adolescents, and is described as the most complete instrument for the evaluation of these patients.^(6,7)^ Based on the above information, the present study aimed to evaluate the overall functional status of pediatric patients after discharge from the PICU, using the FSS scale, to compare functional scores and Pediatric Index of Mortality 2 (PIM2) results in relation to the occurrence of readmission in the PICU and to compare the time of IMV, length of hospitalization and PIM2 results between individuals with different degrees of impairment according to their overall FSS score.

## METHODS

A cross-sectional study was conducted at the PICU and the pediatric hospitalization unit of the *Hospital de Clínicas of Porto Alegre* (HCPA) between March and August 2015. The project was approved by the Research and Ethics Committee of the HCPA under opinion 966.513, and the Informed Consent Form was signed by the parents/guardians. The PICU of the HCPA has 13 beds and admits patients with clinical disorders or after surgical procedures, except trauma situations and cardiac surgery.^(5)^ Patients of both sexes who were discharged from this PICU, over 1 month of age and younger than 18 years old, with a stay in the PICU for a period of ≥ 24 hours were included in the study. Patients dependent on ventilatory technology prior to admission to the PICU, those who were premature (gestational age ≤ 37 weeks) aged ≤ 12 months upon admission to the study and patients readmitted to the PICU within a period of ≤ 24 hours after discharge from the unit were excluded from the study.

The evaluation of the functional status was performed by the FSS (Appendix 1), which was applied to all participants of the study on the first day after discharge from the PICU, always by the same evaluator. This scale has previously been validated for the pediatric population in the English language,^(7)^ and the researchers obtained authorization from the author responsible for its use in this study. The functional scale is composed of six domains: mental status, sensory, communication, motor function, feeding and respiratory. Each domain is categorized from normal (1) to very severe dysfunction (5), and the total score ranges from 6 to 30.

The overall FSS scores are categorized as follows: 6 - 7 normal; 8 - 9, mild dysfunction; 10 - 15, moderate dysfunction; 16 - 21, severe dysfunction; and more than 21 points, very severe dysfunction.^(8)^ The PIM2 scale is predictive of specific mortality in PICU. For its completion, the first value of each clinical variable measured from the time of admission up to 1 hour after arrival in the PICU is recorded.^(9,10)^ This instrument is routinely applied by the medical team of the PICU of the HCPA for all patients admitted to this unit, and their final score is recorded in the electronic medical record.

The clinical and demographic information of the patients, such as age, gender, basic diagnosis, reason for hospitalization, type of hospitalization, origin, length of stay in the PICU and length of IMV were collected from the participants' electronic medical records. The occurrence of death and readmissions was evaluated in October 2015 through the registry of the PICU and electronic medical records.

The estimated sample size was 47 individuals, considering a statistical power of 80%, significance level of 5%, adjusted for a margin of error of 2 points in the FSS scale, and a standard deviation of 4.4, as presented in the original study.^(7)^ WinPEPI software version 11.43 was used to verify correlations of at least 0.4 between the variables of the functional score and the length of hospital stay, mechanical ventilation time and PIM2 results. Quantitative variables were described by the mean and standard deviation when the distribution was symmetrical and by the median and interquartile range for asymmetric distributions, according to the Shapiro-Wilk normality test. For the comparative analyses of IMV time, length of stay and PIM2 results, subjects were divided into two groups, according to the overall FSS score, the first with less functional impairment (normal and mild dysfunction categories), and the second with greater functional impairment (moderate dysfunction, severe dysfunction, and very severe dysfunction categories).

The Mann-Whitney U test was used to compare the functional score according to the occurrence of readmission, differences between the sexes and differences between the groups of major and minor impairment in the overall functional score in relation to the IMV time, length of hospital stay and PIM2 results. A level of 5% was considered significant. Data were analyzed using the Statistical Package for Social Science (SPSS), version 18.0.

## RESULTS

The sample consisted of 50 individuals who were discharged from the PICU of the HCPA and met the criteria for inclusion in the study. The main characteristics of the sample are presented in table 1. The most frequent causes of admission were respiratory failure (40%), postoperative recovery (16%), sepsis and shock (12%) and liver failure (12%).

**Table 1:** Clinical and demographic characteristics of the sample (n = 50)

Variables	
Male	30 (60)
Age (months)	19 [6 - 61]
Origin	
Infirmary	18 (36)
Emergency	9 (18)
Other hospitals	14 (28)
Surgical ward	9 (18)
Type of hospitalization	
Urgent	42 (84)
Elective	8 (16)
Main baseline diagnoses	
Congenital/genetic defects	10 (20)
Neurological	9 (18)
Healthy	8 (16)
Hepatic	7 (14)
Length of stay in the PICU (days)	5 [3 - 12.2]
IMV time (days)	0.75 [0 - 4]
PIM2 (%)	1.13 [0.4 - 4.25]

PICU - pediatric intensive care unit; IMV - invasive mechanical ventilation; PIM2 - Pediatric Index of Mortality 2. Results expressed as N (%) or the median [interquartile 25-75].

The results of the functional evaluation at discharge from the PICU according to the overall FSS and its domain scores are presented in table 2. Regarding the overall functional status, 15 individuals were classified with normal functional status, 7 had mild dysfunction, 20 had moderate dysfunction, 7 had severe dysfunction and 1 individual had very severe dysfunction. A total of 18% of the individuals presented a normal overall FSS (6), and only 6% had an overall FSS ≥ 20, which was considered severe to very severe functional impairment.

**Table 2:** Functional Assessment according to the Functional Status Scale (n = 50)

Domain	
Overall score	11.5 [7 - 15]
Mental status	1 [1 - 2]
Sensory	1 [1 - 1]
Communication	1 [1 - 2]
Motor function	3 [1 - 4]
Feeding	4 [1 - 4]
Respiratory	1 [1 - 2]

Results expressed as the median [interquartile 25-75].

During the study period, 40% of the patients were readmitted to the PICU, and 12% of the patients died. A comparison of the functional score of the FSS and the PIM2 in relation to the occurrence of readmission to the PICU is shown in table 3. No statistically significant difference was observed between the sexes in relation to the overall score and FSS domains.

**Table 3:** Functional Status Scale and Pediatric Index of Mortality 2 functional scores in the readmission and non-readmission groups

Variables	Readmission (n = 20)	Non-readmission (n = 30)	p value
Overall score	13.5 [12 - 16.5]	8 [6 - 12.2]	0.01*
Mental status	1 [1 - 2]	1 [1 - 1.2]	0.158
Sensory	1 [1 - 2]	1 [1 - 1]	0.148
Communication	1.5 [1 - 2]	1 [1 - 1.25]	0.087
Motor function	4 [3 - 4]	1 [1 - 3]	0.01*
Feeding	4 [4 - 4]	1.5 [1 - 4]	0.02*
Respiratory	2 [1 - 2.7]	1 [1 - 2]	0.036*
PIM2	2.7 [0.8 - 15.4]	0.9 [0.3 - 1.9]	0.025*

PIM2 - Pediatric Index of Mortality 2.

* p < 0.05. Mann-Whitney U test. The results are expressed as the median [interquartile 25-75].

A comparison of the PIM2 results, length of hospital stay and IMV time between the groups with the lowest and highest overall functional impairment is shown in table 4.

**Table 4:** Comparison of the Pediatric Index of Mortality 2, length of hospital stay and time of invasive mechanical ventilation between the groups with the lowest and highest overall functional impairment

	Overall FSS score		
	Normal-mild (n = 22)	Moderate-very severe (n = 28)	p value
PIM2 (%)	0.88 [0.40 - 1.31]	2.05 [0.28 - 5.58]	0.182
Length of hospital stay (days)	3.5 [2.00 - 10.25]	6 [4.00 - 19.25]	0.022*
IMV time (days)	0 [0 - 1.25]	2 [0 - 5]	0.044*

FSS - Functional Status Scale; PIM2 - Pediatric Index of Mortality 2; IMV - invasive mechanical ventilation.

* p < 0.05. Mann-Whitney U test. The results are expressed as the median [interquartile 25-75].

## DISCUSSION

In the present study, the majority of subjects presented an overall functional status of moderate dysfunction when evaluated by the FSS on the first day after discharge from the PICU. The greatest functional impairment was demonstrated in the domains of "motor function" and "feeding". For the first time, our study showed that patients who were readmitted to the PICU had worse overall functional status and specifically worse motor function, feeding and breathing scores than those who were not readmitted. However, this same group of patients presented higher predictive mortality rates on the PIM2. Additionally, a longer time of hospitalization in the PICU and greater permanence of IMV were observed in individuals who presented greater overall functional impairment.

The median overall functional score and the percentage of individuals with normal FSS scores and overall FSS scores ≥ 20 found in our population were similar to the results of the original instrument study.^(7)^ The main causes of admission to the PICU, such as respiratory failure, postoperative recovery, sepsis and shock, and liver failure, were similar to those of another study conducted in the PICU of HCPA,^(5)^ likely due to the profile of the patients admitted in this unit.

In the present study, 82% of the patients presented some degree of alteration in the FSS domains after discharge from the PICU (FSS > 6). The greater impairment in the "motor function" and "feeding" domains can be explained by periods of immobility and/or bed restriction during hospitalization in the PICU, which commonly lead to the development of neuromuscular weakness.^(11)^ Regarding feeding dysfunction, we found a high percentage of patients who required enteral nutrition, which is sometimes maintained after discharge from the unit. This result corroborates that found in a study by Pollack et al.,^(8)^ who performed a baseline FSS evaluation using pre-hospitalization retrospective data and, after discharge from the PICU, observed increased morbidity in the "respiratory", "motor function" and "feeding" domains.

The main cause of admission in our study was respiratory failure, but when evaluated after discharge from the PICU, most of the individuals did not present a significant impairment in this domain, which indicates that the patients recover almost completely regarding the ventilatory domain and are weaned from ventilatory support prior to transfer to the pediatric inpatient unit.

An analysis of patients readmitted to the PICU indicated that patients with a worse overall FSS score and impairment in the "motor function", "feeding" and "respiratory" domains at discharge were readmitted to this unit. No other studies have evaluated the relationship between the occurrence of readmissions and functional scores after discharge in the pediatric population. This finding demonstrates that patients with impairment in these functions should be monitored and followed up with greater attention by the multiprofessional team to enhance the recovery of these functions and, possibly, avoid readmission to the PICU. Predictive mortality scores, as an independent risk factor for ICU readmission, have previously been described for adult patients using the Acute Physiologic and Chronic Health Evaluation (APACHE II).^(12,13)^ Patients who were readmitted to the PICU had higher mortality rates based on the PIM2 results at the previous admission, but no studies have evaluated this relationship in the pediatric population.

In contrast to other studies that indicate a relationship between functional status at discharge, assessed by the Pediatric Cerebral Performance Category (PCPC) and the Pediatric Outcome Performance Category (POPC), and predictive mortality rates at admission,^(5,14,15)^ in this study, we did not observe a significant difference in admission PIM2 values, according to the impairment in the overall FSS score presented by individuals at discharge from the PICU*.* This can be explained by Fonseca et al.,^(16)^ who verified that the PIM2 proved to be an inadequate measure to predict the mortality of patients with chronic conditions, which seems to be the case of the population in our study, in which only 16% individuals did not present any health condition prior to hospitalization in the PICU. The authors note the inclusion of few prediction variables to assess these conditions as a limitation of the PIM2,^(16)^ in addition to considering only the first hour of patient hospitalization, as clinical conditions can vary dramatically within the first 24 hours of hospitalization.^(17)^

Fiser et al.^(15)^ verified the association of the motor and cognitive functional assessment scales of the POPC and PCPC with the length of stay in the PICU. A study by Alievi et al.,^(5)^ using the POPC and PCPC, verified a nonlinear increase in a comparison of the functional scores and length of stay in the PICU. In our study, the group of individuals who presented a moderate to very severe overall FSS score had a significantly longer stay in the PICU and a longer IMV time. To date, no other studies have evaluated FSS scores regarding the length of hospital stay and IMV time.

We can note the evaluation of functional status at a single moment at discharge from the PICU as limitation of this study, and it is important to perform other studies to compare FSS scores at admission and discharge from the unit to better indicate new morbidities due to chronic or acute conditions. In addition, validation of the FSS for the Portuguese language should be performed for future studies.

## CONCLUSION

This is the first study using the FSS in the Brazilian population. The results demonstrated a higher prevalence of moderate dysfunction in the overall functional status of the individuals after discharge from the pediatric intensive care unit as well as greater impairment, especially in the physical domains of "motor function" and "feeding", and less impairment in the cognitive domains.

Our findings also demonstrated a relationship between readmission to the pediatric intensive care unit and worse scores on the FSS, higher predictive mortality rates on the PIM2, and a greater overall functional impairment on the FSS, reflecting in significant increase in length of stay in the pediatric intensive care unit and longer invasive mechanical ventilation time.

## APPENDIX

**Appendix 1:** Functional Status Scale

	1	2	3	4	5
	Normal	Mild dysfunction	Moderate dysfunction	Severe dysfunction	Very severe dysfunction
Mental status	Normal sleep/wake; appropriate responsivity	Sleepy but arousable to noise/touch/movement and/or periods of social nonresponsivity	Lethargic and/or irritable	Minimal arousal to stimulus (stupor)	Unresponsive and/or Coma and/or Vegetative
Sensory	Intact hearing and vision and responsive to touch	Suspected hearing or Suspected vision loss	Not reactive to auditory stimuli or Not reactive to visional stimuli	Not reactive to auditory stimuli and Not reactive to visional stimuli	Abnormal response to pain or touch
Communication	Appropriate non-crying vocalizations, interactive facial expressiveness, or gestures	Diminished Vocalization Diminished Facial Expression and/or social responsiveness	Absence of attention getting behavior	No demonstration of discomfort	Absence of communication
Motor function	Coordinated body movements and Normal muscle control and Awareness of action and why it’s being done	1 limb functionally impaired	2 or more limbs functionally impaired	Poor head control	Diffuse Spasticity, Paralysis, Decerebrate/Decorticate Posturing
Feeding	All food taken by mouth with age appropriate help	NPO or need for age -inappropriate help with feeding	Oral and tube feedings	Parenteral Nutrition with oral or tube feedings	All parenteral nutrition
Respiratory	Room air and no artificial support or aids	Oxygen and/or Suctioning	Tracheostomy	CPAP for all or part of the Day and/or Mechanical Ventilator support for part of the day	Mechanical ventilatory support for all of the day and night

Souce: Pollack MM, Holubkov R, Glass P, Dean JM, Meert KL, Zimmerman J, Anand KJ, Carcillo J, Newth CJ, Harrison R, Willson DF, Nicholson C; Eunice Kennedy Shriver, National Institute of Child Health and Human Development Collaborative Pediatric Critical Care Research Network. Functional Status Scale: new pediatric outcome measure. Pediatrics. 2009;124(1):e18-28.^(7)^
